# Dr. Frank Jones

**Published:** 1914-09

**Authors:** 


Dr. Frank Jones, Buffalo, 1903, died in Niagara Falls, August 4, aged 38.

